# The Effect of News Portal Use for Local News on Subjective Well-Being: The Mediating Role of Thwarted Belongingness and the Moderating Role of Self-Esteem

**DOI:** 10.3390/bs16091555

**Published:** 2026-09-02

**Authors:** Doo-Hun Choi

**Affiliations:** Department of Media and Communication, Gachon University, 1342 Seongnamdaero, Sujeong-gu, Seongnam-si 13120, Gyeonggi-do, Republic of Korea; choidoohun1@gmail.com; Tel.: +82-31-750-5242

**Keywords:** news portals, thwarted belongingness, self-esteem, subjective well-being, local news, local community, psychological health

## Abstract

Considering the changing news consumption patterns driven by new media technology and the decline of traditional local news outlets, the current research explored how the use of news portals for local news as emerging channels of news consumption affects the news audiences’ psychological health, particularly their subjective well-being. This study investigated how thwarted belongingness mediates the association between the use of news portals for local news and subjective well-being and how this mediating path varies according to one’s level of self-esteem. Using data from a national online survey in the Republic of Korea, the present research analyzed the direct relationships, mediation effect, and moderated mediation relationship among news portal use for local news, thwarted belongingness, self-esteem, and subjective well-being. Findings show that using news portals for local news has a negative relationship with thwarted belongingness, which is negatively related with subjective well-being. The mediating impact of thwarted belongingness on the association between news portal use for local news and subjective well-being became more pronounced as self-esteem increased. Results suggest that greater news portal use for local news may be associated with higher subjective well-being through lower levels of thwarted belongingness, highlighting the psychological health benefits of local news engagement through digital platforms.

## 1. Introduction

The proliferation of new media technology has dramatically transformed people’s access to, and consumption of, news. In particular, online news portals such as Yahoo News and Google News enable individuals to obtain a diverse range of news content from multiple sources, including traditional mainstream news outlets (e.g., NBC, Reuters, and The New York Times), digital-native news platforms (e.g., BuzzFeed, TechCrunch, or ZDNET), and independent media organizations (e.g., The Texas Tribune or ProPublica) in a single location. This shift is particularly evident in South Korea, where digital news platforms dominate news media. A survey report revealed that almost 70% of the respondents relied primarily on news portals, while the readership of traditional newspapers declined to approximately 10% (Korea Press Foundation, 2023). This sharp contrast highlights the significant transformation of news consumption patterns from traditional offline outlets to digital platforms. These novel news platforms not only streamline news engagement, but also reshape the dynamics of news consumption by altering the traditional gatekeeping role of journalism.

Another significant phenomenon in news media and journalism is the decline of local news media. A growing number of local communities are becoming “news deserts,” leaving local citizens with little or no access to reliable and essential local news and information (Local News Initiative, 2024; Qin, 2024). Despite the shrinking trend of local news outlets (Local News Initiative, 2024), the influence of local news on citizens and society remains vital. For example, empirical evidence has shown that a higher availability of local news outlets is linked to increased voter turnout in local elections (D. Hayes & Lawless, 2021) and that local news media use among citizens contributes to enhancing their political knowledge (Moy et al., 2004). Recent studies have begun to explore how the use of local news affects psychological outcomes in local communities (e.g., D. H. Choi et al., 2021; Nah & Yamamoto, 2019). For instance, local news media use, such as television or newspapers, positively influenced neighborhood belonging and collective efficacy (Nah & Yamamoto, 2019).

However, despite growing interest in the psychological implications of local news use, little research has examined how individuals’ use of news portals to access local news affects their psychological health. Because these portal platforms increasingly serve as primary gateways to news, including local issues, and supplant traditional news distribution channels, it is imperative to understand their role in shaping users’ psychological health. This study examines whether the frequency of accessing local news through news portals is associated with psychological health. Although news portals serve as distinctive platforms, this study measures the frequency of access rather than users’ experiences with specific algorithmic, personalized, or interactive affordances of the news platform. Nevertheless, examining portal-based news access is important, because the platforms constitute a dominant gateway through which South Korean audiences encounter news, including local news (D. H. Kim et al., 2024).

Considering the changing news consumption environment and the impact of local news use, the current study fills this gap by investigating whether news portal use for local news is associated with individuals’ thwarted belongingness, which may in turn be associated with their subjective well-being. Thwarted belongingness indicates one’s perception of social disconnection from other people or social groups, such as communities (Van Orden et al., 2010; Wike et al., 2023). Given the role of local news in cultivating connections with local communities (Hoffman & Eveland, 2010), this study anticipates that the use of news portals for local news is associated with reduced thwarted belongingness. Furthermore, because thwarted belongingness affects psychological health, such as well-being (Yu, 2022), this study also examines whether lower levels of thwarted belongingness are associated with higher subjective well-being, suggesting a potential indirect pathway linking news portal use for local news and subjective well-being through thwarted belongingness.

**Figure 1:**
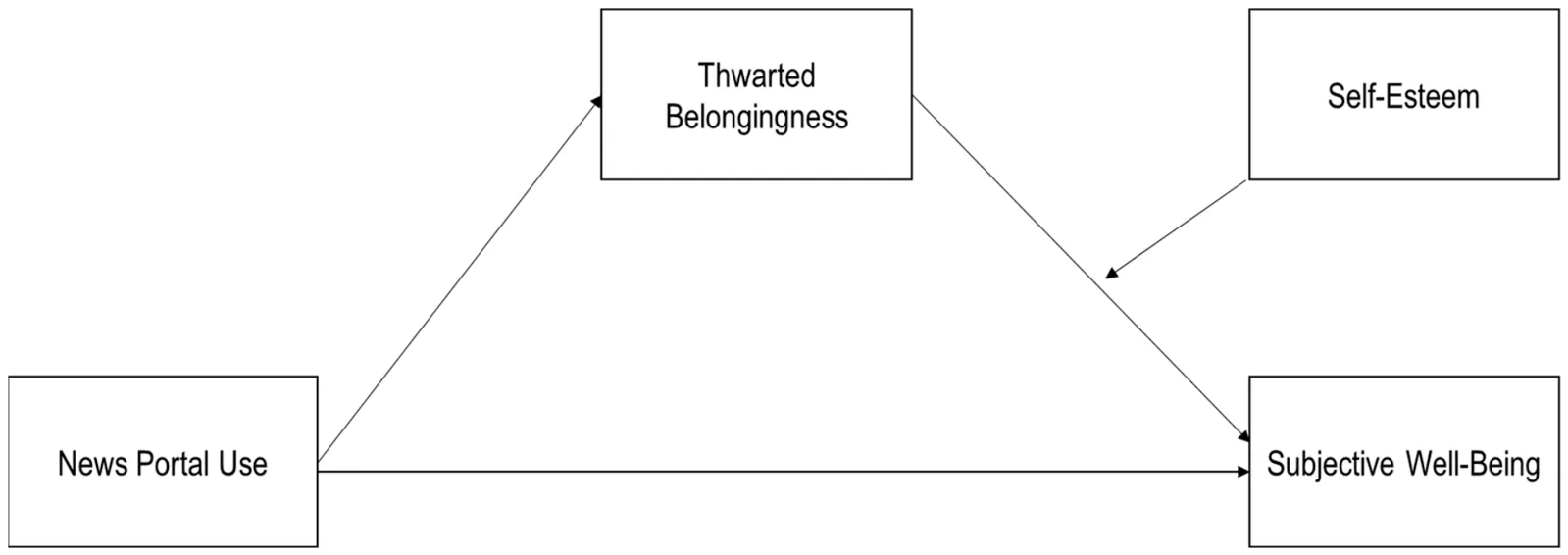
Research model.

Finally, this study examines the moderating role of self-esteem in this mediating relationship. Previous research suggests that individuals’ responses to media use vary according to personal factors such as self-esteem (D. H. Choi & Noh, 2023), and that individual differences in self-esteem may differentially influence subjective well-being (Duy & Yıldız, 2019). Therefore, this study explores whether the indirect association between using news portals for local news and subjective well-being through thwarted belongingness differs depending on individuals’ levels of self-esteem. Figure 1 illustrates the research model describing the hypothesized relationships among the study variables.

## 2. Literature Review

### 2.1. News Portal Use for Local News and Thwarted Belongingness

Online news portals (hereafter, news portals) are digital platforms that aggregate and distribute news content produced by various new media organizations on a single website, such as Yahoo News and Google News (D. H. Kim et al., 2024). News portals do not generate original content themselves. Instead, these platforms function as gateways or intermediaries, utilizing human editors or automated algorithms, and enable users to access diverse news content from multiple publishers free of charge, bypassing individual media outlet websites. In South Korea, owing to high Internet penetration, extensive national wireless broadband infrastructure, and the widespread use of mobile devices, people can easily access news portal platforms, making news portals an important news source for South Korean citizens (Chung et al., 2022; D. H. Kim et al., 2024). In particular, as local news outlets provide news content to news portals such as Naver or Daum (Kang & Choi, 2021), people can conveniently and freely (without cost) access news and information about their local or community issues through these platforms. Moreover, for some local news media that have not fully undergone digital transformation (M. J. Choi et al., 2019), partnerships with news portal platforms can be an effective approach to reach and retain news audiences. For example, some local news outlets have reported that news distribution through news portal platforms increases news article views and enhances audience engagement (D. A. Kim, 2022).

More importantly, this study suggests that news portal use for local news may be associated with thwarted belongingness. Thwarted belongingness is explained as a person’s perception of being socially disconnected or excluded from significant relationships, such as family, friends, or important social communities (Hill et al., 2015; Van Orden et al., 2010). As thwarted belongingness is associated with people’s psychological factors toward their community (e.g., Hill et al., 2017; Liu et al., 2022), previous research has proposed that when arousing community belonging, thwarted belongingness decreases (Chu et al., 2016). In particular, local news can foster community belonging (Hoffman & Eveland, 2010; Zhang, 2020), which is negatively associated with thwarted belongingness. Local news serves as an essential source of important information that helps people manage their daily lives and flourish in their communities (Friedland et al., 2012). Because local news provides people with useful information about their community, it can assist them in developing a sense of belonging to their community. This is partly because local media organizations and news reporters, who are deeply connected to the daily lives and social networks of their communities, can more effectively understand and represent local issues (Qin, 2024). Empirical research has shown that local media use positively affects people’s sense of belonging to their communities (Latikka et al., 2023). Moreover, news portals for local news offer better opportunities to deliver news to a wider audience.

Beyond serving as digital channels for news dissemination, news portals have several technological affordances (Evans et al., 2017) that distinguish them from traditional local news media. Unlike traditional local news outlets, news portals function as platforms that aggregate news stories from multiple news organizations and organize and prioritize news through editorial and algorithmic processes based on users’ interests, offer personalized news recommendations depending on user characteristics and prior patterns of news consumption, and enable interactive engagement through comment and news sharing, restructuring how news is selected, distributed, encountered, and discussed (Kwak et al., 2021). In addition, they allow users to access local news continuously through mobile devices, thereby making local information more visible and routinely accessible.

These affordances of news portals may increase local residents’ exposure to community-related information, facilitate greater awareness of local issues, and increase opportunities for psychological connections with their local communities by shaping how people engage with and experience their local communities. Therefore, news portal use for local news makes users feel informationally embedded in their communities and increases their perceived commonality with other community residents, and they thus may be less likely to perceive themselves as completely disconnected from an important social group. Moreover, the interactive engagement of news portals may foster a sense that local issues are collectively experienced and that users are connected with other community members and local community life, helping to reduce their perceived social isolation. Based on the discussion above, this study argues that news portal use for local news is negatively associated with thwarted belongingness, suggesting the following hypothesis:

**H1.**  *News portal use for local news is negatively associated with thwarted belongingness.*

### 2.2. Thwarted Belongingness and Subjective Well-Being

Subjective well-being is explained as a person’s self-reported evaluation of whether his or her overall life is desirable (Diener et al., 1998). Subjective well-being involves both cognitive judgments and emotional experiences of people’s overall quality of life from their own perspective, representing their psychological health (Larsen & Eid, 2008). Previous studies have demonstrated that subjective well-being is associated with various positive outcomes such as better future health, enhanced social life, and improved future behaviors (Diener, 2012; Diener et al., 2018). For example, a longitudinal study found that individuals with high levels of subjective well-being tend to have longer lifespans than those with lower levels of subjective well-being (Zaninotto & Steptoe, 2019). Additionally, higher subjective well-being is positively associated with better work performance (Diener, 2013). In contrast, lower levels of subjective well-being have been linked to greater suicidal ideation (Lucas-Molina et al., 2018). Thus, it is important for individuals to maintain or enhance their subjective well-being.

As social relationships have a significant impact on subjective well-being (Diener et al., 2018), this study suggests that thwarted belongingness is negatively associated with subjective well-being. According to Baumeister and Leary’s (1995) belongingness theory, individuals tend to have a fundamental and innate desire to create and sustain significant social relationships with others. When this need is thwarted, people may experience psychological distress (Van Orden et al., 2010). Empirical studies have demonstrated that thwarted belongingness is positively associated with psychological health problems, such as depression and suicidal ideation (e.g., Guidry & Cukrowicz, 2016; Venta et al., 2014). Conversely, belonging to a supportive social group or maintaining close interpersonal relationships helps alleviate adverse psychological outcomes such as stress, protects against harmful effects (Y. Jung et al., 2012), and contributes to increased subjective well-being (Romeo et al., 2024). Previous research has shown that belonging to a community has a positive relationship with subjective well-being (Toikko & Pehkonen, 2018). Thus, this study expects that higher levels of thwarted belongingness are associated with lower levels of subjective well-being and proposes the following hypothesis:

**H2.**  *Thwarted belongingness is negatively associated with subjective well-being.*

Based on the discussion above, this study anticipates that thwarted belongingness mediates the relationship between news portal use for local news and subjective well-being. As discussed previously, news portal use for local news is negatively associated with thwarted belongingness. Additionally, thwarted belongingness may negatively affect subjective well-being. Thus, it is likely that news portal use for local news is correlated with thwarted belongingness, which may in turn be related to subjective well-being, indicating the mediating role of thwarted belongingness. Accordingly, this study proposes the following hypothesis:

**H3.**  *Thwarted belongingness mediates the relationship between news portal use for local news and subjective well-being.*

While this study explores the mediating relationship between news portal use for local news and subjective well-being through thwarted belongingness, it also examines the direct relationship between news portal use for local news and subjective well-being. As previous research has found that local news use is positively and directly related to news audiences’ well-being (Park et al., 2024), this study speculates that news portal use for local news may also be directly associated with subjective well-being. In particular, because news portals provide not only information about important issues in local communities but also public spaces for discussing local communities, news portal use for local news as local media can increase community participation (Thorson et al., 2020), which may be positively associated with subjective well-being (Chen & Zhang, 2021). Therefore, this study proposes the following hypothesis:

**H4.**  *News portal use for local news is positively associated with subjective well-being.*

### 2.3. Moderating Role of Self-Esteem

Self-esteem is a person’s perception of self-worth and ability (D. H. Choi, 2025; Rosenberg, 1979). It reflects positive or negative self-evaluations (Morin & Racy, 2021; Rosenberg et al., 1995). Individuals with high self-esteem tend to be more caring and affective than those with low self-esteem (Wood & Forest, 2016). Self-esteem can function as a psychological buffer that protects individuals from negative events, which may increase their subjective well-being (Zeigler-Hill, 2013). Previous studies have demonstrated a positive relationship between self-esteem and subjective well-being (Du et al., 2017; Duy & Yıldız, 2019; Ji et al., 2019). Thus, this study expects self-esteem to have a positive relationship with subjective well-being, and proposes the following hypothesis:

**H5.**  *Self-esteem is positively associated with subjective well-being.*

Finally, this study examines the moderating effect of self-esteem on the relationship between news portal use for local news and subjective well-being through thwarted belongingness. Since the effect of media use on people’s cognitive or behavioral outcomes differs depending on individual variability (e.g., D. H. Choi, 2025; D. H. Choi & Noh, 2023; Y. Kim & Chen, 2015), the impact of news portal use for local news on subjective well-being through thwarted belongingness is likely to vary according to one’s level of self-esteem. As individuals hold different levels of self-esteem (Zeigler-Hill, 2013), thwarted belongingness, which is affected by news portal use for local news, may produce varying levels of subjective well-being. Given this consideration, this study tests a moderated mediation path of news portal use for local news on subjective well-being through thwarted belongingness, which is dependent on levels of self-esteem. This study offers a deeper understanding of how individual differences are associated with the effects of news portal use for local news on subjective well-being through thwarted belongingness. Due to insufficient empirical evidence for this moderated mediation relationship, this study proposes the following research question:

RQ1: How does self-esteem moderate the indirect effect of news portal use for local news on subjective well-being through thwarted belongingness?

## 3. Methods

### 3.1. Data

This study used survey data collected from South Korean adults between 24 November and 10 December 2021. The survey participants were recruited from national online panels administered by a professional survey company in South Korea. A proportionate quota sampling method was used based on age, gender, and regional area to represent the Korean population. An online invitation to participate in the survey was distributed to approximately 120,000 individuals who were randomly selected from the whole panel pool of the research firm. A total of 2223 panelists replied to the email invitation. After excluding incomplete responses, 1000 respondents completed the survey, yielding a completion rate of 45.0%. IRB approval was obtained from Hallym University (IRB No. HIRB-2021-083).

### 3.2. Measures

#### 3.2.1. Subjective Well-Being

The respondents were asked to rate their agreement with the following statements on a 5-point scale ranging from 1 (not at all) to 5 (very much): “In most ways my life is close to my ideal,” “So far I have gotten the important things I want in life,” “The conditions of my life are excellent,” “I am satisfied with my life,” “I am engaged and interested in my daily activities,” and “I feel optimistic and healthy about myself.” The average score of the six items was used to generate an index of subjective well-being (M = 3.11, SD = 0.80, Cronbach’s α = 0.90). The questionnaire items were adapted and modified from earlier works (e.g., Diener et al., 2009a, 2009b).

#### 3.2.2. Thwarted Belongingness

Respondents were asked to score how much they agree with the following statements on a 5-point scale ranging from 1 (strongly disagree) to 5 (strongly agree): “I feel like I belong (reverse coded),” “I am fortunate to have many caring and supportive friends (reverse coded),” “I am close to other people (reverse coded),” “Other people care about me (reverse coded),” and “I feel that there are people I can turn to in times of need (reverse coded).” An index of thwarted belongingness was created by averaging the five items (M = 2.56, SD = 0.71, Cronbach’s α = 0.86). Higher scores indicate greater thwarted belongingness. The questionnaire scales were adapted and modified from prior studies (e.g., Hill et al., 2015; Van Orden et al., 2012).

#### 3.2.3. Self-Esteem

Respondents were asked to indicate their agreement with the following statements on a 5-point scale ranging from 1 (strongly disagree) to 5 (strongly agree): “I take a positive attitude toward myself,” “I feel that I am a person of worth,” “I am satisfied with myself,” and “I feel that I have a number of good qualities.” A composite index of self-esteem was calculated by averaging the four items (M = 3.42, SD = 0.80, Cronbach’s α = 0.87). These measurement questionnaires were adapted and modified from existing research literature (Kong et al., 2012; Rosenberg, 1965).

#### 3.2.4. News Portal Use for Local News

News portal use for local news was measured using a single item by asking respondents to report how frequently they used news portals, such as Daum News or Naver News, to access news about local community issues in the past week. Responses were rated on a 5-point scale (1 = never to 5 = very frequently; M = 3.55, SD = 1.09).

#### 3.2.5. Control Variables

Demographic variables were included such as age (M = 47.49, SD = 13.96), gender (male = 49.8%), education attainment (median = college diploma), and average income per month (median = more than KRW 4,000,000—less than KRW 5,000,000; approximately USD 4000–5000). Local community context variables such as length of residence (i.e., how long respondents have lived in their current residence in months, M = 166.61, SD = 138.60) and community satisfaction (i.e., how satisfied respondents were with their living community on a 7-point scale ranging from 1 = never to 7 = very much, M = 4.64, SD = 1.21) served as controls in the analysis.

### 3.3. Analysis

This study used the SPSS (Version 30.0) PROCESS macro (Version 5.0) to empirically analyze the research hypotheses and questions (A. F. Hayes, 2018). This analytical technique estimates the direct and indirect impacts of independent and dependent variables using ordinary least squares (OLS) regression analysis with a bootstrapping approach (A. F. Hayes, 2018). Specifically, PROCESS Model 14 was employed to examine the direct effect of news portal use for local news, thwarted belongingness, self-esteem, and subjective well-being (H1, H2, H4, and H5), and to test the moderated mediation effect of self-esteem on the indirect relationship between news portal use for local news and subjective well-being through thwarted belongingness (RQ1). In addition, this study applied PROCESS Model 4 to investigate the mediating effect of thwarted belongingness between news portal use for local news and subjective well-being (H3). Control variables, including demographic and community context variables, were contained in the analyses. To determine the significance of the indirect effects, 5000 bootstrap samples with 95% bias-corrected confidence intervals (CIs) were used (A. F. Hayes, 2018). An indirect effect was considered statistically significant if zero was excluded from the 95% confidence interval (A. F. Hayes, 2018). Table 1 presents the correlations among all variables.

## 4. Results

**Figure 2:**
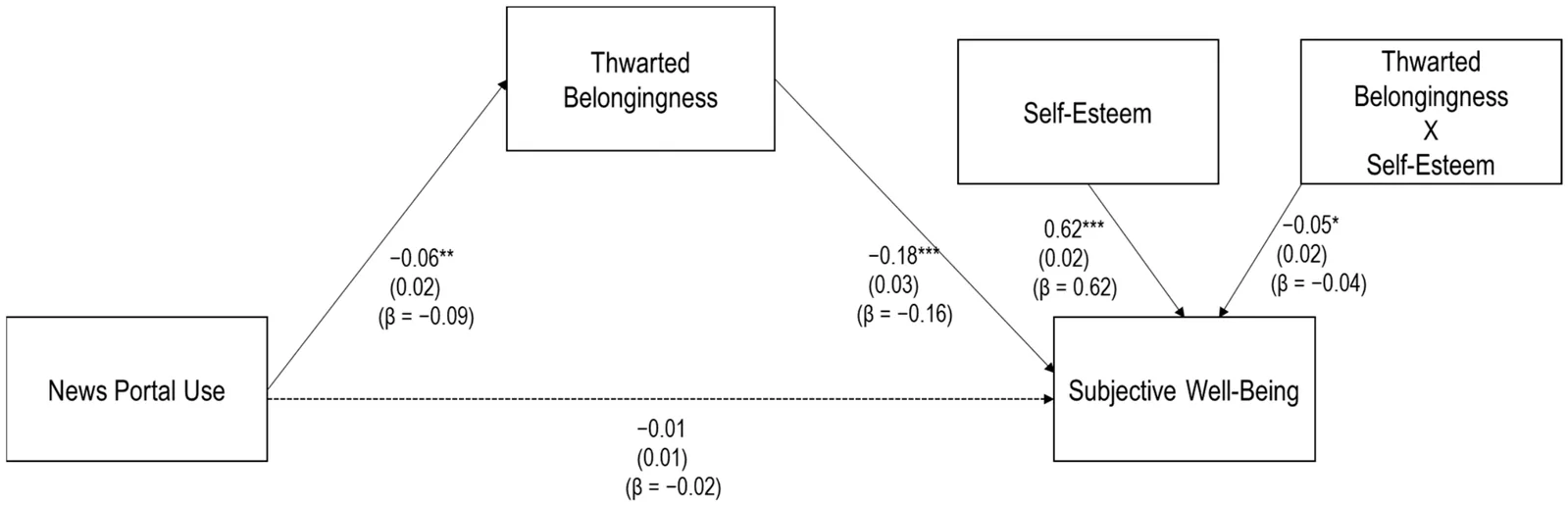
Path coefficients. * *p* < 0.05. ** *p* < 0.01. *** *p* < 0.001.

**Figure 3:**
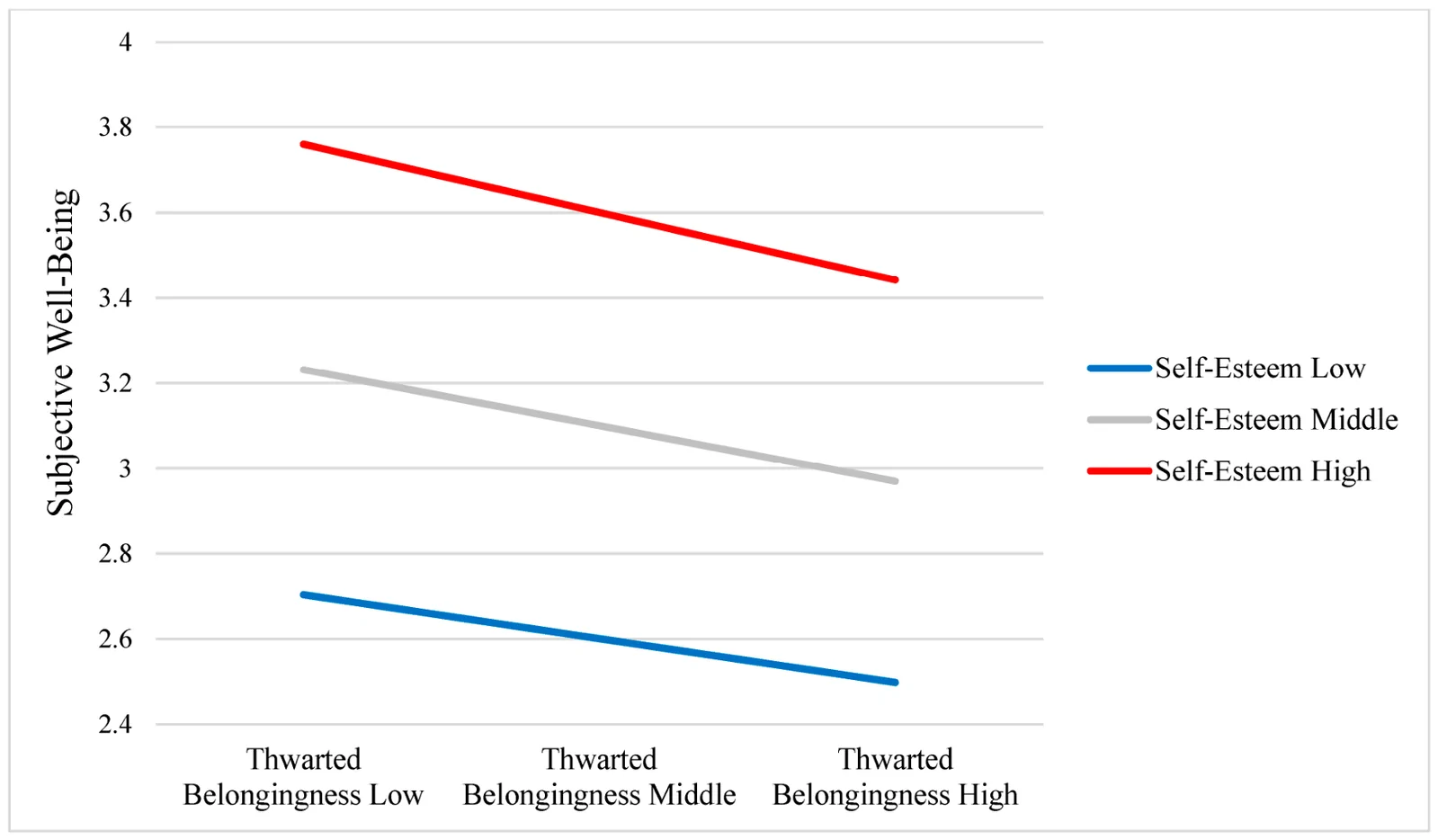
Conditional relationships between thwarted belongingness and subjective well-being across levels of self-esteem. (*Note*: Scale only partially displayed; Low = mean − 1SD, Middle = mean, and High = mean + 1SD).

Figure 2 shows the direct paths among news portal use for local news, thwarted belongingness, self-esteem, and subjective well-being. Supporting H1, this study found that news portal use for local news is negatively associated with thwarted belongingness (*B* = −0.06, *SE* = 0.02, β = −0.09, *p* < 0.01). The analysis also demonstrated a negative association between thwarted belongingness and subjective well-being (*B* = −0.18, *SE* = 0.03, β = −0.16, *p* < 0.001), lending support for H2. However, news portal use for local news did not have a direct relationship with subjective well-being. Thus, H4 was not supported. Additionally, in support of H5, the study found that self-esteem is positively associated with subjective well-being (*B* = 0.62, *SE* = 0.02, β = 0.62, *p* < 0.001). Finally, the interaction between thwarted belongingness and self-esteem on subjective well-being was statistically significant (*B* = −0.05, *SE* = 0.02, β = −0.04, *p* < 0.05). Figure 3 illustrates that the negative association between thwarted belongingness and subjective well-being became stronger with higher levels of self-esteem.

Table 2 presents the results of the mediation analysis. Thwarted belongingness significantly mediated the relationship between news portal use for local news and subjective well-being. Thus, this finding supports H3.

As Table 3 demonstrates, this study found that self-esteem significantly moderates the indirect relationship of news portal use for local news on subjective well-being through thwarted belongingness. Moreover, as presented in Table 4, in response to RQ1, the moderated mediation analysis showed different levels of mediating effects depending on the varying conditions of self-esteem. Specifically, the mediating relationship of news portal use for local news on subjective well-being through thwarted belongingness was significant for low, middle, and high levels of self-esteem. The findings indicate that as self-esteem increases, the mediating effect of news portal use for local news on subjective well-being through thwarted belongingness also becomes greater.

## 5. Discussion

The current study investigated how the use of news portals for local news relates to subjective well-being through thwarted belongingness and whether this indirect relationship differs based on levels of self-esteem. This study yielded two key findings. First, news portal use for local news had a negative relationship with thwarted belongingness, which in turn was negatively related to subjective well-being. Second, this indirect relationship became significantly stronger as self-esteem increased. These findings suggest that greater news portal use for local news may be associated with more favorable psychological outcomes through lower levels of thwarted belongingness.

Specifically, this study found that people who use news portals for local news are more likely to have lower levels of thwarted belongingness. Previous studies have shown that local news use can strengthen community attachment, neighborhood belonging, and collective efficacy by helping residents understand shared local concerns and recognize themselves as members of their local community (Hoffman & Eveland, 2010; Latikka et al., 2023; Nah & Yamamoto, 2019). This study extends the literature by showing that this community-oriented function may also operate when local news is accessible through news portals beyond traditional local media. This finding is particularly relevant in the South Korean news environment, where news portals serve as a major gateway to news content and can broaden access to news information distributed by local media organizations (D. H. Kim et al., 2024).

Two potential mechanisms may explain this association. First, similar to the major role of local news (Y. B. Jung, 2023), news portals for local news may provide opportunities for users to gain a better understanding of their local communities by offering in-depth coverage of local community issues and residents, which could be positively associated with a stronger sense of belonging to their local community. In particular, local news tends to emphasize the shared values and common interests of the local community (Hoffman & Eveland, 2010), and news portal use for local news would play a bridging role between local community residents and local society. By providing broadly important information on local interests and issues to news audiences through theses platforms, news portal use for local news may be associated with lower thwarted belongingness by strengthening users’ perceived connections with their local communities. Second, the interactive features of news portals for local news, such as the function of commenting or sharing local news stories, could be associated with a greater sense of community belonging by engaging in news about the local community. By highlighting local issues, news portals can raise public awareness and encourage public discussion and collaboration among community residents to resolve these issues (Y. B. Jung, 2023). In particular, because news portals enable news audiences to post or share their comments on news content, news portal use for local news can facilitate communication and provide an opportunity to exchange various opinions regarding local community issues, which may correspond to greater perceived engagement with their community through online interactions. Therefore, the observed negative association may be consistent with the possibility that engagement with local news is linked to stronger community connection and lower thwarted belongingness.

The results also confirmed the well-established theoretical link that individuals with higher levels of thwarted belongingness are more likely to report lower levels of subjective well-being. This finding supports the notion of belongingness theory (Baumeister & Leary, 1995), which suggests that failure to satisfy the need for belonging can cause negative health outcomes, such as loneliness or depression. This also resonates with the interpersonal psychological theory of suicide, which explains that thwarted belongingness affects psychological vulnerabilities such as suicidal ideation (Van Orden et al., 2010). This finding can also be interpreted as the reverse: individuals with lower levels of thwarted belongingness are more likely to experience higher levels of subjective well-being. In particular, because subjective well-being plays a protective role against negative mental health states (Li et al., 2025), reducing thwarted belongingness is important. As our study found, lower thwarted belongingness may represent a statistical pathway linking news portal use for local news with higher subjective well-being. This finding indicates that the psychological value of local news lies not simply in information acquisition but also in its potential to reinforce a sense of social and community connection among portal news users for local news. This study highlights an indirect association in which greater news portal use for local news is linked to higher subjective well-being through lower levels of thwarted belongingness.

It is also important to note that news portal use for local news was not directly associated with subjective well-being. This finding differs from previous research reporting a positive association between local news use and audience well-being (Park et al., 2024). One possible explanation is that the psychological benefits of local news use may depend on the mechanisms activated by portal news use rather than arising automatically from exposure itself. In other words, exposure to local news through portals would not be directly associated with individuals’ evaluations of their lives. Instead, when news portal users feel more connected to their local communities and less socially disconnected, their use of news portals for local news may be associated with subjective well-being. This study emphasizes the identification of thwarted belongingness as a potential intervening mechanism between digital local news use and subjective well-being.

Another important finding was that the indirect path to subjective well-being differs according to self-esteem’s level. The estimated indirect association between news portal use for local news and subjective well-being through thwarted belongingness was stronger among individuals with higher self-esteem. One possible interpretation is that individuals with higher self-esteem may be more receptive to community-related information or more likely to integrate such information into positive perceptions of social connectedness. This pattern is consistent with previous studies indicating that self-esteem is positively associated with subjective well-being and can shape how individuals respond to social resources and psychological experiences (Du et al., 2017; Duy & Yıldız, 2019; Ji et al., 2019). This finding indicates that people with higher self-esteem may be more receptive to the community-building aspects of local news, more confident in engaging with local community issues, and more likely to perceive themselves as important members of their local communities, thereby extracting psychological benefits from their media experiences such as reduced thwarted belongingness, which in turn are positively associated with subjective well-being. This interpretation is consistent with the view that self-esteem functions as a psychological resource that influences how people process social experiences and derive their well-being (Zeigler-Hill, 2013). The results also imply that personal differences, such as self-esteem, can generate differing impacts from news media use on psychological health (D. H. Choi, 2025; D. H. Choi & Noh, 2023).

Before drawing conclusions, it is essential to address the limitations of this study. First, because this study used cross-sectional survey data, our empirical findings cannot be used to make causal inferences about the relationships between the observed variables. For instance, although thwarted belongingness is associated with subjective well-being, this finding does not build causality in that thwarted belongingness increases subjective well-being. Individuals with lower subjective well-being may experience greater thwarted belongingness, rather than the reverse. Nevertheless, our research model is grounded in robust theoretical reasoning and supported by empirical findings from earlier studies. Using longitudinal panel data or experimental approaches in future research could help strengthen causal inferences.

Second, the measurement of the news portal use for local news relied on a single item. Although this measure provided a straightforward indicator of how often respondents accessed local news through news portals, it did not capture other dimensions of local news use, including intensity of use (e.g., the amount of time spent or the number of news stories consumed), diversity of use (e.g., the range of local issues or news outlets), or quality and form of engagement (e.g., commenting or sharing). Thus, respondents who report similar frequencies may have differed in the intensity and quality of their local news experiences on news portals. Moreover, because the construct was measured using a single item, internal consistency could not be assessed and retrospective self-reports may have been subject to recall bias and measurement imprecision, potentially influencing the estimated associations. Future research should use multidimensional measures that incorporate the diverse aspects of local news through news portals. Self-reported measures should be supplemented with behavioral indicators, such as digital trace data or news use log data.

Third, the news portal use for local news measure did not distinguish exposure to local news stories from exposure to specific affordances of news portals. This measurement item does not assess respondents’ experiences with algorithmic recommendation, personalization, content aggregation, news commenting, sharing, or other interactive features. Thus, the observed association revealed in this study may not be attributed specifically to general exposure to local news content or the platform characteristics of news portals. Future research should separately measure local news exposure and platform-specific affordances. This measurement approach may help determine whether psychological outcomes arise primarily from local news, platform affordances, or their interactions.

Fourth, although this study controlled for several demographic and community-level characteristics, the observed associations may have been influenced by unmeasured factors. For example, general news use, social connectedness, personality traits, and mental health status may influence the frequency of news portal use for local news and subjective well-being. Moreover, because these factors were not included in the current study, they may represent alternative explanations for the observed relationships. Individuals with stronger social ties, greater community attachment, or better psychological health may be more likely to use news portals for local news. Future studies should incorporate more comprehensive measures to reduce the possibility of omitted variable bias and better isolate the unique contribution of news portal use for local news to subjective well-being.

Finally, the study was conducted exclusively with South Korean respondents, and the external validity of the findings should be considered in relation to the nation’s digital news environment. In South Korea, news portals such as Naver or Daum constitute major gateways through which news audiences obtain news, whereas audiences in other countries tend to rely on direct access to news organizations or social media (Jin, 2022; Reuters Institute, 2022). Thus, the mechanism proposed in the current study may operate differently in other media ecosystems where local news is primarily accessed through local newspapers, broadcasters, websites, social media, or other digital intermediaries. Comparative media-system research suggests that country-specific institutional, market, and political conditions shape the organization, distribution, and consumption of news, contributing to substantial cross-national variations in the platformization of news and patterns of direct access to news websites and applications (Hallin & Mancini, 2004; Nielsen & Fletcher, 2023). Accordingly, the magnitude of the associations observed in this study may not be directly generalizable to other countries with different media consumption environments or local journalistic structures. Future comparative research is needed to explore this mechanism across countries and different local news access channels to identify its contextual boundary conditions.

## 6. Conclusions

Advancements in new media technologies have changed news consumption patterns among news audiences by offering more easily accessible and convenient avenues for obtaining local news, particularly through news portals. Given recent concerns about the decline of traditional local news media, the current research examined the effects of news portal use for local news as a newly emerging local news consumption channel on news audiences’ psychological health. The findings showed that greater news portal use for local news was associated with lower thwarted belongingness and, indirectly, with higher subjective well-being. This indirect association was stronger with higher self-esteem levels.

Our study has important implications for research and practice regarding news media and local communities. From a research perspective, this study introduced and empirically tested a novel conceptual model established in the pre-existing literature to investigate the associations between news portal use for local news, thwarted belongingness, subjective well-being, and self-esteem. While previous studies have largely focused on the political or civic implications of traditional local news outlets, relatively few have attempted to explore how news portal use for local news can be associated with psychological health outcomes such as subjective well-being, particularly through the pathway to thwarted belongingness. Our study extends the literature by identifying thwarted belongingness as a potential psychological correlate linking news portal use for local news with subjective well-being. Thus, future studies should further investigate how news portal use for local news is associated with community belonging and news audiences’ psychological health across diverse cultural contexts and with broader measures of news engagement in the evolving media environment.

From a practical perspective, maintaining accessible digital channels for local news may be relevant to efforts aimed at strengthening residents’ connections with local communities. Facilitating public access to local news through news portals may provide residents with opportunities to access community-relevant information. Future research should examine whether individuals with lower self-esteem experience the community-related aspects of local news differently and whether additional forms of social support are needed for them to derive similar psychological relevance from local news engagement. Finally, it will be necessary to implement a policy that supports the inclusion of small-scale local news media organizations in news portal platforms, enabling them to distribute their news content broadly and helping diverse local community residents to access local news more easily and conveniently. Supporting the inclusion of small local news outlets in major news portals could broaden the distribution of hyperlocal content and facilitate access to community-relevant information, particularly in underserved areas, which would help mitigate the informational and psychological health gaps resulting from the decline of traditional local news media. With the ongoing evolution of the new media landscape, understanding the multifaceted effects of local news is important for strengthening civic life and individual psychological health.

## Figures and Tables

**Table 1:** Correlation coefficients among variables (*N* = 1000).

Variable	1	2	3	4	5	6	7	8	9	10
1. Subjective well-being	—									
2. News portal use for local news	0.167 ***	—								
3. Thwarted belongingness	−0.614 ***	−0.171 ***	—							
4. Self-esteem	0.774 ***	0.200 ***	−0.622 ***	—						
5. Age	−0.010	−0.047	0.019	0.076 *	—					
6. Gender	−0.067 *	−0.026	0.028	−0.063 *	0.040	—				
7. Education	0.084 **	0.047	−0.071 *	0.096 **	−0.171 ***	−0.199 ***	—			
8. Income	0.252 ***	0.146 ***	−0.277 ***	0.259 ***	0.020	−0.055	0.265 ***	—		
9. Length of residence	−0.027	−0.009	0.024	−0.043	0.213 ***	0.014	−0.063 *	0.047	—	
10. Community satisfaction	0.461 ***	0.146 ***	−0.392 ***	0.371 ***	0.064 *	−0.015	0.007	0.187 ***	0.041	—

*Note.* Values are Pearson’s zero-order correlation coefficients. * *p* < 0.05. ** *p* < 0.01. *** *p* < 0.001.

**Table 2:** Indirect effect of news portal use on subjective well-being through thwarted belongingness.

Indirect Path	Estimate	*SE*	CI
News portal use → thwarted belongingness → subjective well-being	0.032	0.011	0.010 to 0.054
	0.044	0.015	0.014 to 0.075

*Note*: Unstandardized (above) and completely standardized (below) regression coefficients and corresponding standard errors are reported; CIs are bias-corrected 95% confidence intervals for the indirect effects (bootstrap *N* = 5000).

**Table 3:** Index of moderated mediation.

	Index	*SE*	CI
Self-esteem	0.002	0.001	0.0001 to 0.0073

**Table 4:** Conditional indirect effects of news portal use on subjective well-being through thwarted belongingness moderated by levels of self-esteem.

Moderator	Condition	Estimate	*SE*	CI
Self-esteem	Low	0.008	0.003	0.002 to 0.016
Self-esteem	Middle	0.010	0.004	0.003 to 0.020
Self-esteem	High	0.013	0.005	0.003 to 0.025

*Note*: Unstandardized regression coefficients and corresponding standard errors are reported; conditions for the moderator (self-esteem) are the mean (Middle) and one standard deviation below (Low) and above (High) the mean. CIs are bias-corrected 95% confidence intervals for the indirect effects (bootstrap *N* = 5000).

## Data Availability

The datasets presented in this article are not readily available due to privacy and ethical restrictions. Requests to access the datasets should be directed to the corresponding author, Doo-Hun Choi, upon reasonable request.
